# Association between Nucleoside and Nucleotide Reverse Transcriptase Inhibitor Use and Primary Open-Angle Glaucoma Risk in *All of Us*

**DOI:** 10.1016/j.ophtha.2025.06.014

**Published:** 2025-06-21

**Authors:** Kenneth Pham, Fangming Jin, Roy Lee, Isabel Di Rosa, Rebecca Salowe, Gui-Shuang Ying, Joan M. O’Brien

**Affiliations:** 1 Penn Medicine Center for Genetics of Complex Disease, Department of Ophthalmology, University of Pennsylvania, Philadelphia, Pennsylvania.; 2 Department of Ophthalmology, University of Pennsylvania, Philadelphia, Pennsylvania.; 3 Center for Preventive Ophthalmology and Biostatistics, Perelman School of Medicine at the University of Pennsylvania, Philadelphia, Pennsylvania.

**Keywords:** *All of Us*, Nucleoside and nucleotide reverse transcriptase inhibitors, Primary open-angle glaucoma

## Abstract

**Purpose:**

To assess the association between the systemic use of nucleoside and nucleotide reverse transcriptase inhibitors (NRTIs) and primary open-angle glaucoma (POAG).

**Design:**

Retrospective cohort study.

**Participants:**

Individuals aged ≥40 years with linked electronic health record (EHR) data in the National Institutes of Health (NIH) *All of Us* dataset. Participants with a diagnosis of POAG before the use of NRTIs were excluded.

**Methods:**

A cohort of 1:10 NRTI users to nonusers was created using a propensity score matching design, considering age, race, sex at birth, human immunodeficiency virus (HIV) diagnosis, hepatitis B diagnosis, and family history of POAG. A multivariable logistic regression model was used to adjust for residual imbalance. Sensitivity analyses were performed for self-reported eye doctor visits.

**Main Outcome Measure:**

Diagnosis of POAG.

**Results:**

Among the 305 441 *All of Us* participants aged ≥40 years with a linked EHR, we identified 718 individuals (0.24%) with NRTI use, excluding participants with a diagnosis of POAG before NRTI exposure. The rate of POAG in the NRTI users was 4.32% (N = 31). The rate of POAG in the propensity score–matched control group (N = 7180) was 2.00% (N = 144). Use of NRTI was associated with an increased risk of POAG (odds ratio [OR], 2.21; 95% confidence interval [CI], 1.48–3.28; *P* < 0.001). When adjusting for residual imbalance of family history of POAG, HIV diagnosis, and hepatitis B diagnosis, use of any NRTIs remained significantly associated with an increased risk of developing POAG (OR, 1.84; 95% CI, 1.22–2.77; *P* = 0.004). After matching and adjusting for self-reported eye doctor visits, NRTIs remained significantly associated with POAG risk (OR, 2.30; 95% CI, 1.07–4.96; *P* = 0.033).

**Conclusions:**

Use of NRTIs was associated with a higher risk of POAG with propensity score matching for covariates and adjusting for residual imbalances.

**Financial Disclosure(s):**

The author(s) have no proprietary or commercial interest in any materials discussed in this article.

The leading cause of irreversible blindness worldwide is glaucoma, of which the most common form is primary open-angle glaucoma (POAG).^1-4^ Often, because of the disease’s asymptomatic nature in early stages, patients remain undiagnosed until late in the disease course when there are no available treatments to reverse the glaucomatous damage. This highlights a need to identify risk factors for POAG to allow for earlier diagnosis and sight-saving intervention.

Previous studies have identified systemic drug exposures that are associated with POAG risk.^5-7^ We study the use of nucleoside and nucleotide reverse transcriptase inhibitors (NRTIs) because of their known association with mitochondrial toxicity through the inhibition of mitochondrial polymerase gamma.^8,9^ Mitochondrial dysfunction has been proposed to contribute to glaucomatous damage and has been visualized in mouse models of glaucoma.^10-12^ Human genetic studies have identified mitochondrial mutations associated with POAG.^13-18^ Moreover, elevated levels of flavoprotein fluorescence, a marker of mitochondrial dysfunction, have been detected in the eyes of individuals with POAG compared with control eyes.^19,20^ Thus, NRTI use may increase the risk of POAG.

A retrospective analysis of an Australian national prescription database previously found that anti-retroviral therapy for human immunodeficiency virus (HIV) is associated with an increased prevalence in use of intraocular pressure–lowering medications in men aged 30 to 49 years.^21^ Anti-retroviral therapy exposure included not only NRTIs but also protease inhibitors, non-nucleoside reverse transcriptase inhibitors, integrase inhibitors, fusion inhibitors, and entry inhibitors. Therefore, there is a need to clarify the association between NRTIs specifically and risk of glaucoma pathogenesis.

Our study aims to investigate the potential risk of POAG and NRTI exposure through its analysis of these variables among the participants of the National Institutes of Health (NIH) *All of Us*^22^ study.

## Methods

### *All of Us* Cohort

To evaluate the association between NRTI exposure and POAG risk, the data (v8) from the NIH *All of Us* study^22^ were analyzed. Participants aged ≥40 years with a linked electronic health record (EHR) were included. Following guidelines put forth by the Office for Human Research Protections, the *All of Us* Institutional Review Board reviewed the protocol, the informed consent, and other participant-facing materials for the *All of Us* Research Program. All participant data were deidentified, and the Declaration of Helsinki was followed.

Participants were classified for different diagnoses based on Systematized Nomenclature of Medicine terms. For POAG, “primary open angle glaucoma,” “bilateral primary open angle glaucoma,” “primary open angle glaucoma of left eye,” and “primary open angle glaucoma of right eye” were considered. For HIV diagnosis, “acute HIV infection,” “asymptomatic human immunodeficiency virus infection,” “human immunodeficiency virus infection,” and “symptomatic human immunodeficiency virus infection” were considered. For hepatitis B diagnosis, “acute type B viral hepatitis,” “chronic type B viral hepatitis,” “chronic viral hepatitis B without delta-agent,” “type B viral hepatitis,” and “viral hepatitis B without hepatic coma” were considered. A positive family history for POAG was based on the survey question “Including yourself, who in your family has had glaucoma?” with the answer of any first-degree relative being sufficient. Additionally, eye conditions other than POAG were investigated. For angle-closure glaucoma, SNOMED concept ID 374028 was used; for cataract, SNOMED concept ID 4088106 was used.

### NRTI Exposure

Exposure for NRTIs was extracted using Anatomical Therapeutic Chemical Classification System designation J05AF. Only participants with a record of the prescription being dispensed in pharmacy were considered as NRTI exposed. Participants with POAG diagnosed before the start of NRTIs were excluded from the analyses.

For the identified participants with NRTI exposure, a propensity score matching strategy was used to create a balanced control group without NRTI exposure using greedy matching with a caliper distance of 0.2 of the standard deviation of the propensity score (caliper: 0.0094). For each NRTI user, 10 matched controls were analyzed. Covariates used for propensity score matching included age, race, sex at birth, HIV diagnosis, hepatitis B diagnosis, and family history of POAG. Additionally, a sensitivity analysis was performed wherein only individuals with a self-reported eye doctor visit were included. The additional covariate of eye doctor visits was used in addition to the aforementioned covariates for propensity score matching. Responses to the survey question “What is the total number visits to an eye doctor that you made in the last 12 months?” were used to identify individuals for inclusion and to match for propensity score matching. Finally, sensitivity analysis with only individuals who have HIV was performed.

### Geographic Distribution Analysis

The geographical data available in the *All of Us* study are the first 3 digits of the participant’s zip code. The NRTI users per 3-digit zip code were counted. Then, similar to the study by Tran et al,^23^ publicly available data from the Centers of Medicare and Medicaid Services and American Community Survey (2023) were used to map ophthalmologists by their associated 5-digit zip codes. Zip codes for the ophthalmologists were truncated to 3-digit zip codes. Finally, the NRTI users per 3-digit zip code and ophthalmologists per 3-digit zip code were compared after normalization per 100 000 residents using the American Community Survey (2023) to tabulate the population per 3-digit zip code.

### Statistical Analysis

Statistical significance was assessed with a Wald test from the logistic regression model. Next, accounting for covariates with residual imbalance (standard mean difference > 0.1), multivariable logistic regression modeling was performed to account for HIV diagnosis, hepatitis B diagnosis, family history of POAG, and adjusted odds ratio (OR) and its 95% confidence interval (CI). For the OR for self-reporting an eye doctor visit within the last year, a Fisher exact test was used. These statistical analyses were performed using Python in the *All of Us* workbench.

## Results

### NRTI Use and POAG Risk

**Figure 1.**
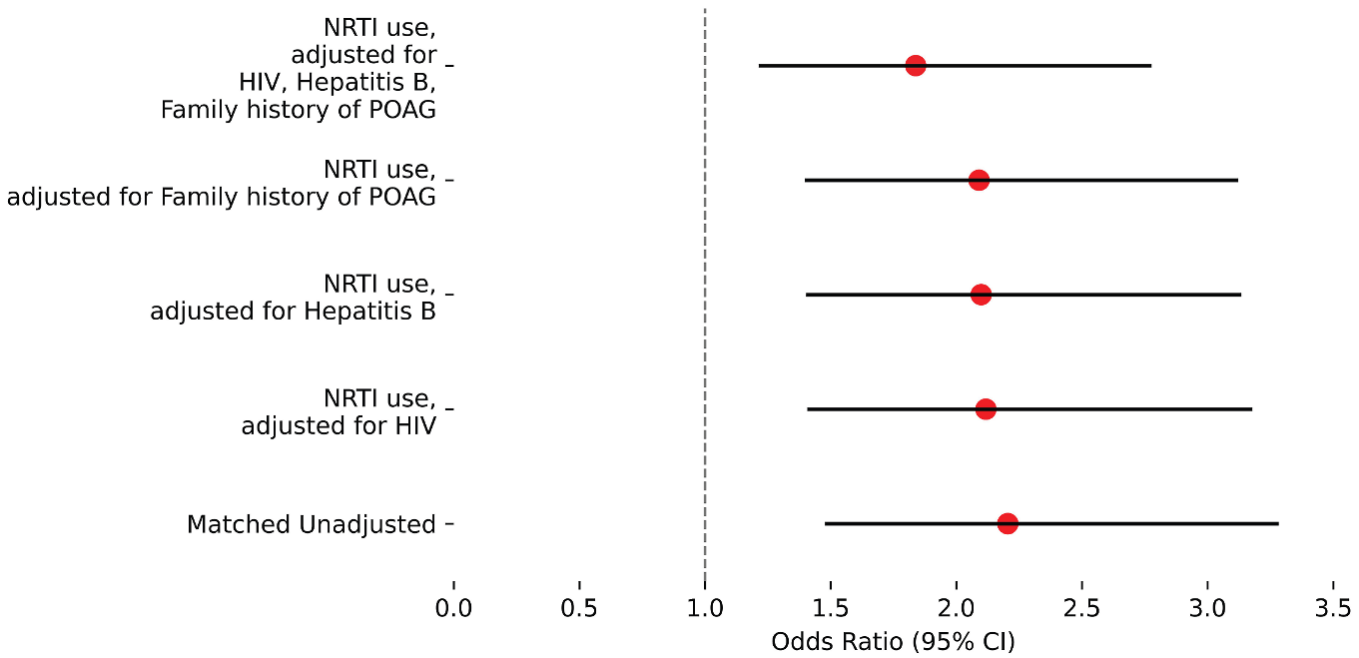
Odds ratios (ORs) for primary open-angle glaucoma (POAG) in nucleoside and nucleotide reverse transcriptase inhibitor (NRTI) users. Variables used for propensity score matching: age, race, sex at birth, HIV diagnosis, hepatitis B diagnosis, and family history of POAG. CI = confidence interval; HIV = human immunodeficiency virus.

Among the NIH *All of Us* participants aged ≥40 years and with a linked EHR, we identified 718 individuals (0.24%) with NRTI use, excluding participants with a diagnosis of POAG before NRTI use. We then constructed a control group with propensity score matching of age, race, sex at birth, HIV diagnosis, hepatitis B diagnosis, and family history of POAG, yielding 7898 total participants. Table 1 details the characteristics of the cohort. In the NRTI-exposed group, the rate of POAG was 4.32% (N = 31). In the control group, the rate of POAG was 2.00% (N = 144). Nucleotide reverse transcriptase inhibitor use was associated with an increased risk of POAG (OR, 2.21; 95% CI, 1.48–3.28; *P* < 0.001) (Fig 1, “NRTI use, unadjusted”).

We noted that, after propensity score matching, a few covariates were imbalanced: family history of POAG, HIV diagnosis, and hepatitis B diagnosis (Table 1). We proceeded to adjust for each of these covariates individually and all 3 of these covariates together (Fig 1, Table 2). After the latter adjustment, NRTI use was still associated with an increased risk of POAG (OR, 1.84; 95% CI, 1.22–2.77; *P* = 0.004) (Fig 1, “NRTI use, adjusted for HIV, hepatitis B, and family history of POAG”).

Next, to address whether NRTI users have more frequent eye exams, which could increase the chances of a POAG case being captured and diagnosed, we performed three additional analyses. First, using the survey data in *All of Us*, we found that 29.8% of NRTI users in *All of Us* had self-reported eye doctor visits compared with 29.9% of NRTI nonusers. We found that NRTI users did not self-report more eye doctor visits than NRTI nonusers (OR, 1.00; 95% CI, 0.85–1.17; *P* = 1). Second, we performed a sensitivity analysis with a cohort of only individuals with self-reported eye doctor visits and included the self-reported number of visits for propensity score matching. We found that the associations between NRTI use and POAG risk survived with only individuals with eye doctor visits (OR, 2.30; 95% CI, 1.07–4.96; *P* = 0.033). Last, we investigated NRTI use and risk of two other ocular conditions: cataracts and angle-closure glaucoma. Our analyses revealed no association between NRTI use and cataract risk (OR, 1.02; 95% CI, 0.72–1.45; *P* = 0.91) nor with angle-closure glaucoma risk (OR, 1.29; 95% CI, 0.27–6.12; *P* = 0.75).

Next, we considered whether HIV infection, including its duration and severity, could be a potential confounder. Thus, we performed a sensitivity analysis by repeating the analysis in a solely HIV^−^ cohort (i.e., HIV ^−^ NRTI users vs HIV^−^ nonusers). We found 275 HIV^−^ NRTI users and performed propensity score matching with HIV^−^ controls. With this HIV^−^ cohort (N = 3025), we did not find statistically significant evidence of an association between NRTI use and POAG risk (OR, 1.37; 95% CI, 0.60–3.11; *P* = 0.46).

Finally, we addressed if NRTI users were preferentially located with better glaucoma detection. When we compared the geographic distribution of NRTI users and ophthalmologist per capita across the United States, we found no association between density of NRTI users and ophthalmologists (Fig S2, available at www.aaojournal.org).

## Discussion

We assessed for potential associations between the use of NRTIs and POAG risk. Using data from the nationwide, diverse NIH *All of Us* study, we found that the use of NRTIs was associated with an increased risk of POAG.

Our results align with a previous retrospective analysis of an Australian prescription database that demonstrated an increased prevalence in intraocular pressure–lowering medications use in men aged 30 to 49 years on antiretroviral therapy, which included NRTIs and other drugs for the treatment of HIV.^21^ In our study, we solely investigated NRTIs, allowing us to refine our question to focus on these medications. Additionally, we explicitly considered HIV as a covariate. Previously, a large-scale retrospective analysis identified associations between systemic medications and POAG.^7^ That study did not identify an association between NRTI use and POAG risk. However, the study was limited in its number of cases and controls with NRTIs: Of the 6310 POAG cases, only 1 was an NRTI user, and of the 30 650 controls, only 10 were NRTI users. Thus, leveraging the size of the *All of Us* cohort enables us to present evidence that NRTI use, specifically, is associated with an increased risk of POAG.

A possibility that could explain the association between NRTI use and POAG risk is that increased medical surveillance associated with NRTI use could allow greater opportunity for POAG diagnosis, although we have performed multiple analyses to detect such a mechanism. We first found that NRTI users were not more likely to report a visit to the eye doctor. Then, we repeated our analyses leveraging *All of Us* survey with only individuals with self-reported eye doctor visits in the last year. The association of NRTI use and POAG diagnosis survived. Moreover, analyses for cataracts and angle-closure glaucoma did not reveal an association between NRTI use and those other eye conditions. Together, these results do not find evidence that higher POAG rates observed among NRTI users can be due to merely more examination of the eyes.

### Limitations

Our study is limited in its retrospective nature. We have addressed potential confounders by using a propensity score–matching strategy and then adjusting for covariates with residual imbalances. On the basis of the present study, we cannot speak to the mechanism through which NRTI exposure is linked with increased POAG risk. Because of the low number of HIV^−^ NRTI users in our study, we are not equipped to rule out the possibility that HIV infection, including its duration and severity, contributes to the association between NRTI use and POAG risk we describe. As individuals who use NRTIs prophylactically increases, future analyses will shed more light on this point. Finally, although we posit that NRTI-associated mitochondrial toxicity may be a contributing factor, future work will elucidate the molecular basis of NRTI-associated POAG risk.

## Conclusions

Our study shows that NRTI exposure is associated with a higher incidence of POAG. Future prospective studies of individuals at risk of POAG can stratify by NRTI exposure to contribute evidence to the question of causation.

## Supplementary Material

Supp Figure

## Figures and Tables

**Table 1.** Characteristics of the Variables Used for Propensity Score Matching to Create a Cohort of Nucleoside and Nucleotide Reverse Transcriptase Inhibitor Users and Nonusers Matched 1:10

	NRTI Users	Controls
N	718	7180
Age (mean ± SD)	60.7 ± 10.8	60.7 ± 10.8
Sex at Birth, N (%)		
Male	563 (78.4%)	5630 (78.4%)
Female	138 (19.2%)	1380 (19.2%)
Race, N (%)		
White	363 (50.6%)	3630 (50.6%)
Black or African American	190 (26.5%)	1900 (26.5%)
>1 population	38 (5.3%)	380 (5.3%)
Asian	21 (2.9%)	210 (2.9%)
Other answers or declined to answer	106 (14.8%)	1060 (14.8%)
Family and Medical History, N (%)		
Family history of POAG	66 (9.2%)	342 (4.8%)
HIV diagnosis	443 (61.7%)	2574 (35.8%)
Hepatitis B diagnosis	119 (16.6%)	924 (12.9%)

HIV = human immunodeficiency virus; NRTI = nucleoside and nucleotide reverse transcriptase inhibitor; POAG = primary open-angle glaucoma; SD = standard deviation.

**Table 2.** Odds Ratio of POAG with NRTI Exposure in the Propensity Score Matching Cohort of NRTI Users and Nonusers, Unadjusted and Adjusted for Residual Imbalanced Covariates

	OR	95% CI	*P* Value
Matched, unadjusted	2.21	1.48–3.28	<0.001
Adjusted for HIV	2.12	1.41–3.17	<0.001
Adjusted for hepatitis B	2.10	1.41–3.13	<0.001
Adjusted for POAG family history	2.10	1.40–3.11	<0.001
Adjusted for HIV, hepatitis B, and POAG family history	1.84	1.22–2.77	0.004

CI = confidence interval; HIV = human immunodeficiency virus; NRTI = nucleoside and nucleotide reverse transcriptase inhibitor; OR = odds ratio; POAG = primary open-angle glaucoma.

## Abbreviations and Acronyms:

CI: confidence interval
EHR: electronic health record
HIV: human immunodeficiency virus
NIH: National Institutes of Health
NRTI: nucleoside and nucleotide reverse transcriptase inhibitor
OR: odds ratio
POAG: primary open-angle glaucoma
