# Supplementary material for: Association between Nucleoside and Nucleotide Reverse Transcriptase Inhibitor Use and Primary Open-Angle Glaucoma Risk in All of Us
Source: Ophthalmology. Author manuscript; Available in PMC 2025 Sep 24. (PMC12453943; doi:10.1016/j.ophtha.2025.06.014)
Supplement: Supp Figure [file NIHMS2111442-supplement-Supp_Figure.pdf]

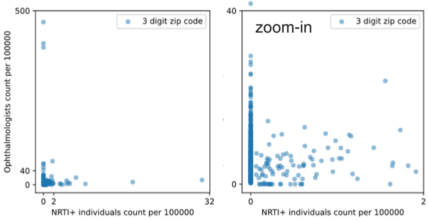

Supplementary Figure 2. Comparison of the distributions of NRTI users and ophthalmologists across the United States
